# Periodontal health and metabolic status of type 1 diabetic children and adolescents

**DOI:** 10.3389/fdmed.2025.1454008

**Published:** 2025-11-18

**Authors:** L. D. J. Scholtz-Evans, A. Jeftha, F. Kimmie-Dhansay, E. W. Zöllner

**Affiliations:** 1 Department of Oral Medicine and Periodontology, Faculty of Dentistry, The University of the Western Cape, Cape Town, South Africa; 2 Department of Community Dentistry, Faculty of Dentistry, The University of the Western Cape, Cape Town, South Africa; 3 Department of Paediatrics, Paediatric Endocrinology, Faculty of Health Sciences, Stellenbosch University, Cape Town, South Africa

**Keywords:** diabetes mellitus (DM), periodontal disease (PD), diabetic type 1 (T1DM), diabetic type 2 (T2DM), glycated haemoglobin (HbA1c), basic periodontal examination (BPE)

## Abstract

**Background:**

Several meta-analyses of children and adolescents with Type 1 diabetes mellitus (T1DM) have shown that periodontal disease (PD) is linked to metabolic control. In South Africa, the prevalence of PD and its impact on T1DM children is however unknown. This study aimed to assess the prevalence of PD in T1DM children and adolescents attending the Paediatric Diabetic Clinic at Tygerberg Hospital and to assess the impact of metabolic control on the periodontal status.

**Methods:**

A cross-sectional study was conducted to assess the periodontal status of T1DM patients. A basic periodontal examination (BPE) was performed and information on the HbA1c level, type and duration of T1DM, age, sex, BMI percentile, and pubertal status was gathered from patient records. A logistic regression model was used to identify associations between periodontal health status and risk factors.

**Results:**

All 169 T1DM participants [median age 11.0 (9.0, 14) years] presented with PD. Based on BPE codes, 124 (73%) had gingivitis and 45 (27%) had periodontitis. The median HbA1c was significantly higher in the periodontitis group [12.6% (IQR: 10.4–13.6)] compared to the gingivitis group [8.9% (IQR: 8.1–9.9)] was statistically significant *p* = <0.001. Age was identiﬁed as risk factors (OR = 1.23, 95% CI: 1.1–1.4; *p* = 0.002), with children in the periodontitis group being older [13.0 (10, 15) years] than those with gingivitis [11.0 (8.0, 13.5) years; *p* < 0.001].

Additionally, the periodontitis group had a lower median BMI percentile [59 (IQR: 29–78)] than the gingivitis group [74 (IQR: 42–92); *p* < 0.001].

**Conclusions:**

Besides the limitations inherent to the study design, every child and adolescent with T1DM presented with periodontal disease. Older age and poorer metabolic control were significantly associated with increased odds of periodontal disease, highlighting a strong link between metabolic control and periodontal health in this population. Longitudinal studies or clinical trials with adequate sample sizes are recommended. These findings underscore the need to intensify both diabetes management and dental care through integrated, long-term approaches.

## 1 Introduction

Diabetes Mellitus (DM), a chronic metabolic disorder, represents a major global public health issue, affecting approximately 425 million individuals worldwide; with the estimated prevalence of Type 1 Diabetes Mellitus (T1DM) globally around 20 million people (1, 2). In South Africa, the incidence of T1DM in children aged 0–14 years is approximately 0.8 per 100,000 population (1–4). T1DM is most commonly diagnosed in childhood or adolescence but can occur at any age (1, 5). T1DM arises from the destruction of pancreatic beta cells, leading to an absolute deficiency of insulin and subsequent hyperglycaemia (5–9). This autoimmune-mediated destruction is triggered by environmental factors and influenced by genetic predisposition (5–9).

Periodontal disease (PD) is an inflammatory condition affecting the supporting structures of the teeth, collectively known as the periodontium (10). It is primarily initiated by the dental biofilm, which triggers an abnormal immune response in the host, ultimately leading to the degradation of periodontal tissues (10, 11). PD encompasses two primary disease entities: gingivitis and periodontitis. Gingivitis, an inflammatory disease limited to the free gingival tissue, always precedes periodontitis. If left untreated, gingivitis can progress to involve all periodontal tissues, resulting in periodontitis (10–12). Periodontitis is characterized by the loss of attachment, particularly involving gingival connective tissue, alveolar bone, and the periodontal ligament. Advanced, unmanaged periodontitis leads to tooth mobility and eventual tooth loss (13, 14).

In children, gingivitis is more prevalent than periodontitis (14, 15), with dental biofilm- induced gingivitis being the most common form (14, 15). The accumulation of biofilm provokes an immune response, leading to gingival inflammation (16). In cases where gingival inflammation occurs with minimal biofilm presence, systemic conditions such as diabetes mellitus may be implicated (13, 16). Gingivitis is reversible with improved oral hygiene practices, however, in more severe cases, professional dental cleaning to remove biofilm becomes necessary (13). If gingivitis remains untreated, it may progress to early- onset periodontitis in susceptible individuals, extending inflammation to the periodontal ligament and alveolar bone (14).

Host susceptibility is a crucial factor in the development of periodontitis, with DM recognized as a significant risk factor due to its shared inflammatory pathogenesis (17, 18). Under hyperglycaemic conditions, the formation of advanced glycation end products (AGEs) activates inflammatory pathways, contributing to gingivitis and the development of periodontitis (18, 19). AGEs accumulate in periodontal tissues through non-enzymatic glycation, intensifying tissue destruction by attracting inflammatory cells and increasing cytokine production (20, 21). Additionally, periodontitis may act as an endogenous source of AGEs, with inflamed periodontal tissues and pathogens such as Tannerella forsythia contributing to elevated local and systemic AGE levels (22). This bidirectional relationship complicates glycaemic control in diabetic patients, perpetuating a cycle of inflammation and tissue damage (23). Effective control of glycated haemoglobin (HbA1c) levels in children with T1DM is therefore essential to prevent progression from gingivitis to periodontitis (18, 24). HbA1c level control serves as a key modifying factor in both DM and PD prognoses. This is reflected in the 2018 classification of periodontal and peri-implant diseases and conditions (25–27).

Recent systematic reviews and meta-analyses conducted in 2015, 2021 and 2023 have examined the periodontal health of children and adolescents with T1DM (12, 28–31). These studies consistently report that T1DM children and adolescents have poorer periodontal health, strongly correlated with their metabolic control, as indicated by HbA1c % levels.

Compared to non-diabetic peers, individuals with T1DM exhibit significantly higher levels of plaque accumulation, bleeding on probing, clinical attachment loss, and periodontal probing depths (28–31). The studies emphasize the importance of monitoring periodontal risk markers in this population and highlight specific contributing factors such as puberty and a high body mass index (BMI) (24, 29, 32–34). Puberty, in particular, is identified as an influential factor in the development of PD in both diabetic and non-diabetic children (35).

Despite the growing international evidence, the prevalence and impact of PD in children with T1DM in South Africa remain unknown. To address this gap, a study was conducted at Tygerberg Hospital to determine the prevalence of PD among children and adolescents with T1DM and to assess the relationship between HbA1c levels and periodontal health status.

## 2 Methods

### 2.1 Participants

A descriptive cross-sectional study was conducted among children and adolescents diagnosed with T1DM to determine their periodontal health and metabolic status. The study population consisted of a convenient sample of patients, who attended the Paediatric Out-patient Diabetic Clinic at Tygerberg Hospital. The required sample size was determined as 182 which was based on a 5% level of significance, a power of 80% and a guestimate odds ratio of 1.7 when assessing whether there was an association between periodontal diagnosis (gingivitis) VS healthy subjects in participants who were not metabolically controlled. Patients between the ages of five and nineteen years were invited to participate in the study. All patients and their parents or guardians received an information sheet detailing the purpose and procedures of the study in a language that the patient understood (Afrikaans, English, and isiXhosa). Participation was entirely voluntary, and refusal to participate, for any reason, did not result in any penalties or discriminatory treatment. Data was collected from 180 patients from June 2019 until July 2020. Participants were excluded if they were 6yrs with no permanent molars or older than 19 or if they had T2DM or were undergoing active orthodontic treatment or had physical disabilities or cognitive impairments that could interfere with oral hygiene practices. A total of 11 patients were excluded from the study: Ten patients were due to a diagnosis of T2DM and one due to an extremely low body mass index (BMI). Patients identified during the study as requiring dental care were appropriately referred to the Oral Health Centre at Tygerberg, the Faculty of Dentistry of the University of the Western Cape, for clinical management.

### 2.2 Ethical permissions

The Biomedical Research and Ethics Committee (BMREC) of the University of the Western Cape granted ethical clearance to conduct the study, and the Ethics registration number is BM19/9/5. In accordance with the Declaration of Helsinki of the World Medical Association (36), informed consent was obtained from all participants and their parents or legal guardians. Participants and their parents or guardians were assured that their rights to privacy, confidentiality, and complete anonymity were upheld.

### 2.3 Data collection

A research questionnaire with closed-ended questions was used to record the type and duration of DM, HbA1c levels, age, sex, body mass index (BMI) percentile (33), and pubertal status. The HbA1c level was taken on the day of the basic periodontal examination (BPE), was recorded. Failing this, the HbA1c level recording on the day closest to the day of the BPE was documented. Puberty was diagnosed by a qualified specialised physician in paediatric endocrinology and was defined by Tanner stages 2–5 and pre-puberty was defined by Tanner stage 1 and was recorded as “yes” or “no” (37). The BMI was calculated in STATA 17 using the CDC BMI-for-age growth charts. The z score was then transformed into a percentile, and the percentiles were categorized into weight status categories as described in Table 1 (33).

**Table 1:** BMI categories (33).

Weight status category	Percentile range
Underweight	Less than the 5th percentile
Healthy Weight	5th percentile to less than the 85th percentile
Overweight	85th to less than the 95th percentile
Obesity	Equal to or greater than the 95th percentile

### 2.4 Oral examination

A Basic Periodontal Examination (BPE) was performed to assess the prevalence of periodontal disease, following the guidelines of the British Society of Periodontology (38–40). The BPE is a simple, reliable screening tool designed to detect disease affecting the free gingival structures and to determine the presence of periodontal disease in patients (39). The examination was conducted using a World Health Organization (WHO) 621 periodontal probe, which is specifically designed for BPE screening. This probe features a 0.5 mm ball end, a black band between 3.5 mm and 5.5 mm, and additional markings at 8.5 mm and 11.5 mm (39, 40). The principal researcher, a qualified dentist familiar with this protocol through daily clinical use, performed all examinations to ensure consistency and reduce inter-operator variability. During the examination, each fully erupted permanent tooth was assessed for bleeding on probing, the presence of plaque and calculus, and periodontal pocket depth. This was done by dividing the mouth into 6 sextants and using the WHO/BPE probe with a light force to probe around each permanent tooth. Periodontal tissues of all permanent teeth were examined to ensure that the highest score in the sextant was recorded before examining the next sextant. The BPE scores codes 0, 1, 2, 3, 4 and * was utilized (38–41). Table 2 presents the BPE scoring system as described by Corbet (2012), which aligns clinical conditions with corresponding BPE score codes. While the original classification assigned the asterisk (*) to indicate “attachment loss of 7 mm at any site,” this descriptor is no longer used in current clinical practice; instead, the asterisk now indicates furcation involvement only. Corbet's classification served as a reference to guide periodontal clinical diagnosis during the study (41).

**Table 2:** BPE codes and their clinical descriptions*Corbet 2012 (41).

Code	Examination findings	Clinical condition
0	No pockets exceeding 3 mm, no calculus or overhangs, and no bleeding on gentle probing.	Clinical Health
1	The coloured band remains completely visible, indicating no pockets exceeding 3 mm, and no calculus or overhangs, but bleeding is present on gentle probing.	Gingivitis
2	The coloured band remains completely visible, indicating no pockets exceeding 3 mm, calculus or other plaque-retentive factors were found at or below the gingival margin, with or without bleeding on probing.	Gingivitis with plaque retention factors
3	The coloured band on the probe remains partially visible when inserted into the deepest pocket, indicating pocket depths greater than 3.5 mm but less than 5.5–6 mm.	Periodontitis (Mild)
4	The coloured band on the probe is covered by gingiva, indicating a pocket at least 6 mm depth.	Periodontitis (Moderate to severe)
*	Attachment loss at any site is 7 mm or greater and furcation involvement	Periodontitis (Severe)

### 2.5 Statistical analysis

Data analyses were performed using the RStudio 2025.05.0 + 496 “Mariposa Orchid” release (f0b76cc00df96fe7f0ee687d4bed0423bc3de1f8, 2025-05-04) for windows Mozilla/5.0 (Windows NT 10.0; Win64; ×64) AppleWebKit/537.36 (KHTML, like Gecko) RStudio/2025.05.0 + 496 Chrome/132.0.6834.210 Electron/34.5.1 Safari/537.36, Quarto 1.6.42.

The data was presented as number (percentage), or median [Interquartile Range] or 95% CI [confidence interval] depending on the distribution of data. For categorical data, a Chi- square or Fisher's exact test was utilized. For continuous data, a Wilcoxon rank sum test or Mann–Whitney *U* test was used to demonstrate any differences between the two groups. A simple and adjusted logistic regression model was used to determine the associations between risk indicators and periodontal disease. All data was significant at a *p*-level of less than 0.05.

## 3 Results

### 3.1 Demographic variables and periodontal status

Demographic variables of this study included 169 participants with T1DM, with a mean age of 11.0 years and a median disease duration of 4 years. The sample consisted of nearly equal numbers of males and females, with about half of the participants in puberty. The median BMI percentile was 68.0 [IQR: 40, 91] (Table 3). The median HbA1c level was 9.6% [IQR: 8.3–10.9]. Fourteen participants (8%) had good metabolic control (HbA1c ≤ 7.5%), while 155 (92%) had poor control (HbA1c > 7.5%) (Table 3).

**Table 3:** Demographic variables and periodontal status.

Demographic variables and periodontal status		Total *n* (%)*
Age (years)	Median (IQR)	11.0 (9.0, 14.0)
Sex	Male	84 (50%)
	Female	85 (50%)
Puberty	Yes	81 (48%)
	No	88 (52%)
Number of years since diagnosis	Median (IQR)	4.00 (2.00, 6.00)
HbA1c (%)	Median (IQR)	9.6 [8.3–10.9]
BMI Percentile	Median (IQR)	68 (40, 91)
Metabolic Control	Controlled	14 (8%)
	Uncontrolled	155 (92%)
Periodontal Status	**Gingivitis**	124 (73.4)
	BPE Code 1	20
	BPE Code 2	104
	**Periodontitis**	45 (27.0)
	BPE Code 3	

* Unless otherwise specified.

Periodontal status was assessed using the BPE code. Participants with a BPE code of 1 (11.8%, *n* = 20) or 2 (61.5%, *n* = 104) meaning they had bleeding on probing, plaque and calculus retaining factors with probing depths below 3.5 mm were categorized as Gingivitis and 124 (73.4%) were included in this definition. The rest of the participants (27.0%, *n* = 45) were categorized as having Periodontitis with a BPE of 3 (Table 3) and had probing depths of above 3.5 mm up to 5.5 mm, bleeding on probing, plaque and calculus retaining factors.

### 3.2 Associated risk factors for periodontal status

HbA1c levels were significantly higher in participants with periodontitis compared to those with gingivitis, and those with periodontitis were also significantly older (Table 4). Both age and HbA1c levels were significantly associated with periodontal status (*p* < 0.001), whereas metabolic control showed a strong influence on periodontal status (*p* = 0.002) (Table 4). Children with gingivitis tended to have higher BMI percentiles than those with periodontitis (*p* = 0.036), while sex, duration of T1DM disease, and puberty showed no significant influence whether the children or adolescents presented with gingivitis or periodontitis (Table 4).

**Table 4:** Risk indicators by periodontal status.

Risk indicators by periodontal status		Gingivitis 124 (73.0%)	Periodontitis 45 (27.0%)	*p*-value
Age (years)	Median [IQR]	11.0 (8.0, 13.5)	13.0 (10.0, 15.0)	<0.001*
Sex	Male	64 (76%)	20 (24%)	0.5
	Female	60 (71%)	25 (29%)	
Puberty	No	64 (79%)	17 (21%)	0.12
	Yes	60 (68%)	28 (32%)	
Number of years since diagnosis	Median [IQR]	4.00 (2.00, 6.00)	5.00 (2.00, 7.00)	0.3
HbA1c (%)	Median [IQR]	8.9 [8.1–9.9]	12.6 [10.4–13.6]	<0.001*
BMI percentile	Median [IQR]	74 (42, 92)	59 (29, 78)	0.036*
Metabolic control	Controlled	14 (11%)	0 (0%)	0.002*
	Uncontrolled	110 (89%)	45 (100%)	

* Statistically significant.

Multivariate logistic regression analysis (Table 5) confirmed that both age and HbA1C% remained significant contributors to the development of periodontitis. Each 1-unit increase in HbA1c% is associated with 2.38 times higher odds of having periodontitis. Additionally, older children exhibited 23% higher odds of having periodontitis than their younger counterparts. BMI percentile was borderline associated with periodontitis (*p* = 0.06). Contrary to expectations, pubertal status was not associated with periodontitis.

**Table 5:** Uni- and multivariable logistic regression model for periodontitis.

Risk factor	Unadjusted OR (95% CI)	*p*-value	Adjusted OR (95% CI)	*p*-value
Age (years)	1.21 (0.9–1.35)	<0.001*	1.23 (1.1–1.41)	0.002*
Mean BMI (percentile)	0.3 (0.1–0.93)	0.037*	0.32 (0.09–1.05)	0.06
Duration of DM (years)	1.04 (0.93–1.15)	0.5	0.95 (0.83–1.07)	0.4
Puberty-yes	1.76 (0.88–3.58)	0.11	1.06 (0.48–2.39)	0.9
Sex-male	1.33 (0.67–2.67)	0.4	1.29 (0.62–2.71)	0.5
HbA1c (%)	2.28 (1.81–3.01)	<0.001*	2.38 (1.83–3.23)	<0.001*

Adjusted for Age, HbA1c, BMI, and duration of DM and puberty.

* Statistically significant.

## 4 Discussion

Periodontal disease (PD) is a prevalent oral condition that significantly contributes to the global burden of chronic disease (42). In SA, data on the prevalence of periodontal disease among children and adolescents with T1DM is limited. This study aimed to explore the relationship between metabolic control and periodontal health status in patients with T1DM and the findings will contribute to the increasing evidence linking PD severity with glycaemic control.

Research shows that patients with T1DM exhibit compromised periodontal status, including increased gingival inflammation, plaque accumulation, probing pocket depth, and clinical attachment loss compared to healthy controls (43–48). Poor glycaemic control in T1DM patients is associated with more severe periodontal destruction, with a higher prevalence of periodontitis observed in those with poor metabolic control (45, 47, 48). Longer disease duration and the presence of diabetic complications also correlate with increased periodontal inflammation (29, 43).

Notably, all participants in this study presented with a PD; 73% were with gingivitis, while 27% had periodontitis. Internationally, similar patterns have been observed: in Turkey, 69% of children aged 5–9 years and 83.7% of those aged 10–14 years with T1DM had gingivitis (49), while in Malaysia, a case-control study of 32 diabetic patients found that 96.8% had PD, with only 6.2% diagnosed with periodontitis. These findings collectively highlight the high prevalence of PD among children and adolescents with T1DM across different populations (50).

Poor metabolic control was seen in both patients with gingivitis and periodontitis in the current study; however, it was substantially and significantly worse in the latter group. This could imply that periodontitis children were generally poorer controlled than the gingivitis children or that the very high HbA1c levels in the periodontitis group are the manifestation of a vicious cycle between hyperglycaemia and inflammation. Other authors refer to this vicious cycle as the “two-way” or “bidirectional relationship” between T1DM and PD (17, 31), which is evident in the findings of the current study that children and adolescents with T1DM exhibit 2.38-times higher odds of having periodontitis for every 1% increment in HbA1c levels.

The relationship between diabetes duration and PD has also been explored in studies from Serbia and India, where only a weak correlation was observed (32, 51). Some evidence suggests that periodontal pocket depth may increase after five years of diabetes duration (50). In current study, although the median diabetes duration among those with periodontitis was four years, this factor was not significantly associated with PD. A prospective longitudinal study design, tracking periodontal status in newly diagnosed children over time, may be more effective in elucidating this potential relationship.

Associated risk factors influence PD, with puberty recognized as a contributing factor in its progression (35, 52), in the present study, children with gingivitis were significantly younger typically in early adolescence compared to those with periodontitis, who were in late adolescence. Despite this age difference, puberty itself was not found to be significantly associated with the presence of PD in this study population.

Sex-based differences in the prevalence of PD among individuals with T1DM have been observed in various populations. For instance, in Mexico, males exhibited a higher prevalence of PD than females, particularly in relation to diabetes duration and metabolic control (51). In contrast, studies on non-diabetic adolescents in the UK revealed that while females had a higher incidence of dental caries, no sex-based differences were found in periodontal status (53). Research from India suggests that sex may influence immune responses related to periodontal health, with males showing a greater risk for chronic periodontitis (35). However, the current study did not identify any significant association between sex and the presence of PD among children and adolescents.

Elevated body mass index (BMI) has been well-documented as a risk factor for PD in both children and adults (34, 54, 55). In the current study, logistic regression analysis revealed a borderline association between mean BMI percentile and periodontitis suggesting that lower BMI may play a potential role in periodontal susceptibility among children with T1DM. Interestingly, the median BMI percentile of children with gingivitis was significantly higher than that of those with periodontitis, as determined by the Mann–Whitney *U* test. This observation aligns with J.M. Goodson's hypothesis that high BMI may contribute to the development of gingivitis (56). However, evidence from other studies in diabetic children and adolescents has shown that increased BMI is also correlated with a higher number of teeth exhibiting attachment loss, a key indicator of periodontitis (20, 34, 55). Together, these findings suggest that the relationship between BMI and periodontal outcomes may be complex, with both low and high BMI potentially influencing disease presentation in different ways.

## 5 Limitations and strengths

The study population hailed from a tertiary endocrinology department, where the majority of participants demonstrated suboptimal metabolic control, potentially introducing selection bias. Furthermore, the cross-sectional design limited causal inference, and the absence of a non-diabetic control group restricted the estimation of excess risk attributable to T1DM.

Periodontal diagnoses were based on the Basic Periodontal Examination (BPE), as resource constraints precluded comprehensive periodontal charting and radiographic evaluation. This reliance on BPE may have resulted in diagnostic overestimation, while the lack of radiographic imaging hindered the exclusion of pseudo pockets. Consequently, these limitations may have influenced the estimated prevalence of periodontitis, and the reported figures should be interpreted with caution.

Potential misclassification of puberty may have occurred due to inaccuracies in medical records, with a possibility that more participants were in established puberty than documented. Time and workload constraints also limited clinician involvement, necessitating reliance on existing records.

Behavioural risk factors, such as smoking, were not assessed, as these are not routinely evaluated in paediatric clinics. Including such variables in future prospective studies would enhance the robustness of findings. Lastly, the study was underpowered to detect statistically significant differences in BMI percentiles, thereby limiting the interpretability of this variable, although observed trends may still hold clinical relevance.

Despite these limitations, the study offers valuable insights into the association between T1DM and periodontal health in paediatric patients. Its strengths include a reasonable single-centre sample size (*n* = 169) and the application of logistic regression to evaluate key associations.

## 6 Conclusions and recommendations

All children and adolescents with DM who attended the Paediatric Diabetes Clinic at Tygerberg Hospital presented with some degree of periodontal disease (PD). Age and metabolic control emerged as significant risk factors for PD, with a positive correlation between diabetic control and periodontal disease. Among the participants, 28% were found to have a BPE score of 3, which may be a clinical indicator of periodontitis. This group of patients tended to be older with a lower BMI compared to those who were diagnosed with gingivitis. A longitudinal prospective studies or clinical trials with adequate sample sizes, including periodontal parameters and radiographs, would provide stronger evidence and help guide interventions more effectively in this patient population.

## Data Availability

The raw data supporting the conclusions of this article will be made available by the authors, without undue reservation.
