# Supplementary material for: Periodontal health and metabolic status of type 1 diabetic children and adolescents
Source: Front Dent Med. 2025 Nov 18;6:1454008. doi: 10.3389/fdmed.2025.1454008 (PMC12669169; doi:10.3389/fdmed.2025.1454008)
Supplement: Supplementary file 1 [file Datasheet2.pdf]

Supplementary Material

2.3 Data Collection

Table 1. *BMI categories(33)*

Weight Status Category	Percentile Range
Underweight	Less than the 5th percentile
Healthy Weight	5th percentile to less than the 85th percentile
Overweight	85th to less than the 95th percentile
Obesity	Equal to or greater than the 95th percentile

2.4 Oral Examination

Table 2. *BPE codes and their clinical descriptions*Corbet 2012(41)*

Code	Examination findings	Clinical condition
**0**	*No pockets exceeding 3 mm, no calculus or overhangs, and no bleeding on gentle probing.*	*Clinical Health*
**1**	*The coloured band remains completely visible, indicating no pockets exceeding 3 mm, and no calculus or overhangs, but bleeding is present on gentle probing.*	*Gingivitis*
**2**	*The coloured band remains completely visible, indicating no pockets exceeding 3 mm, calculus or other plaque-retentive factors were found at or below the gingival margin, with or without bleeding on probing.*	*Gingivitis with plaque retention factors*
**3**	*The coloured band on the probe remains partially visible when inserted into the deepest pocket, indicating pocket depths greater than 3.5 mm but less than 5.5–6 mm.*	*Periodontitis (Mild)*
**4**	*The coloured band on the probe is covered by gingiva, indicating a pocket at least 6 mm depth.*	*Periodontitis (Moderate to severe)*
*	*Attachment loss at any site is 7 mm or greater and furcation involvement*	*Periodontitis (Severe)*

3 Results

3.1 Demographic variables and Periodontal status:

Table 3: Demographic variables and periodontal status

Demographic variables and periodontal status		Total n (%) *
**Age (years)**	median (IQR)	11.0 (9.0, 14.0)
**Sex**	Male	84 (50%)
	Female	85 (50%)
**Puberty**	Yes	81 (48%)
	No	88 (52%)
**Number of years since diagnosis**	median [IQR]	4.00 (2.00, 6.00)
**HbA1c (%)**	median [IQR]	9.6 [8.3 to 10.9]
**BMI Percentile**	median [IQR]	68 (40, 91)
**Metabolic Control**	Controlled	14 (8%)
	Uncontrolled	155 (92%)
**Periodontal Status**	**Gingivitis**	124 (73.4)
	BPE Code 1	20
	BPE Code 2	104
	**Periodontitis**	45 (27.0)
	BPE Code 3	

** Unless otherwise specified*

3.2 Associated risk factors for periodontal status:

Table 4: Risk indicators by periodontal status

Risk indicators by periodontal status		Gingivitis 124 (73.0%)	Periodontitis 45 (27.0%)	p-value
Age (years))	median [IQR]	11.0 (8.0, 13.5)	13.0 (10.0, 15.0)	<0.001*
Sex	Male	64 (76%)	20 (24%)	0.5
	Female	60 (71%)	25 (29%)	
Puberty	No	64 (79%)	17 (21%)	0.12
	Yes	60 (68%)	28 (32%)	
Number of years since diagnosis	median [IQR]	4.00 (2.00, 6.00)	5.00 (2.00, 7.00)	0.3
HbA1c (%)	median [IQR]	8.9 [8.1 to 9.9]	12.6 [10.4 to 13.6]	<0.001*
BMI Percentile	median [IQR]	74 (42, 92)	59 (29, 78)	0.036*
Metabolic Control	Controlled	14 (11%)	0 (0%)	0.002*
	Uncontrolled	110 (89%)	45 (100%)	

Table 5: *Uni- and Multivariable logistic regression model for periodontitis.*

Risk factor	Unadjusted OR (95% CI)	p-value	Adjusted OR (95% CI)	p-value
**Age (years)**	1.21 (0.9 to 1.35)	<0.001*	1.23 (1.1 to 1.41)	0.002*
**Mean BMI (percentile)**	0.3 (0.1 to 0.93)	0.037*	0.32 (0.09 to 1.05)	0.06
**Duration of DM (years)**	1.04 (0.93 to 1.15)	0.5	0.95 (0.83 to 1.07)	0.4
**Puberty-Yes**	1.76 (0.88 to 3.58)	0.11	1.06 (0.48 to 2.39)	0.9
**Sex-Male**	1.33 (0.67 to 2.67)	0.4	1.29 (0.62 to 2.71)	0.5
**HbA1c (%)**	2.28 (1.81 to 3.01)	<0.001*	2.38 (1.83 to 3.23)	<0.001*

Adjusted for Age, HbA1c, BMI, and duration of DM and puberty
** Statistically significant*
