# Supplementary material for: Periodontal health and metabolic status of type 1 diabetic children and adolescents
Source: Front Dent Med. 2025 Nov 18;6:1454008. doi: 10.3389/fdmed.2025.1454008 (PMC12669169; doi:10.3389/fdmed.2025.1454008)
Supplement: Supplementary file 2 [file Datasheet1.docx]

Supplementary Material

**2.3 Data Collection**

***Table 1****. BMI categories(33)*

| **Weight Status Category** | **Percentile Range** |
| --- | --- |
| Underweight | Less than the 5^th^ percentile |
| Healthy Weight | 5^th^ percentile to less than the 85^th^ percentile |
| Overweight | 85^th^ to less than the 95^th^ percentile |
| Obesity | Equal to or greater than the 95^th^ percentile |

**2.4 Oral Examination**

***Table 2****. BPE codes and their clinical descriptions*Corbet 2012(41)*

| **Code** | **Examination findings** | **Clinical condition** |
| --- | --- | --- |
| ***0*** | *No pockets exceeding 3 mm, no calculus or overhangs, and no bleeding on gentle probing.* | *Clinical Health* |
| ***1*** | *The coloured band remains completely visible, indicating no pockets exceeding 3 mm, and no calculus or overhangs, but bleeding is present on gentle probing.* | *Gingivitis* |
| ***2*** | *The coloured band remains completely visible, indicating no pockets exceeding 3 mm, calculus or other plaque-retentive factors were found at or below the gingival margin, with or without bleeding on probing.* | *Gingivitis with plaque retention factors* |
| ***3*** | *The coloured band on the probe remains partially visible when inserted into the deepest pocket, indicating pocket depths greater than 3.5 mm but less than 5.5–6 mm.* | *Periodontitis (Mild)* |
| ***4*** | *The coloured band on the probe is covered by gingiva, indicating a pocket at least 6 mm depth.* | *Periodontitis (Moderate to severe)* |
| ******* | *Attachment loss at any site is 7 mm or greater and furcation involvement* | *Periodontitis (Severe)* |

1. **Results**
   1. **Demographic variables and Periodontal status:**

***Table 3******:*** *Demographic variables and periodontal status*

| *Demographic variables and* *periodontal status* | | Total n (%) * |
| --- | --- | --- |
| Age (years) | median (IQR) | 11.0 (9.0, 14.0) |
| Sex | Male | 84 (50%) |
|  | Female | 85 (50%) |
| Puberty | Yes | 81 (48%) |
|  | No | 88 (52%) |
| Number of years since diagnosis | median [IQR] | 4.00 (2.00, 6.00) |
| HbA1c (%) | median [IQR] | 9.6 [8.3 to 10.9] |
| BMI Percentile | median [IQR] | 68 (40, 91) |
| Metabolic Control | Controlled | 14 (8%) |
|  | Uncontrolled | 155 (92%) |
| Periodontal Status | **Gingivitis**  BPE Code 1  BPE Code 2 | 124 (73.4)  20  104 |
|  | **Periodontitis**  BPE Code 3 | 45 (27.0) |

** Unless otherwise specified*

- 1. **Associated risk factors for periodontal status:**

***Table 4:*** *Risk indicators by periodontal status*

| Risk indicators by periodontal status | | Gingivitis 124 (73.0%) | Periodontitis 45 (27.0%) | p-value |
| --- | --- | --- | --- | --- |
| **Age (years))** | median [IQR] | 11.0 (8.0, 13.5) | 13.0 (10.0, 15.0) | <0.001* |
| **Sex** | Male | 64 (76%) | 20 (24%) | 0.5 |
|  | Female | 60 (71%) | 25 (29%) |  |
| **Puberty** | No | 64 (79%) | 17 (21%) | 0.12 |
|  | Yes | 60 (68%) | 28 (32%) |  |
| **Number of years since diagnosis** | median [IQR] | 4.00 (2.00, 6.00) | 5.00 (2.00, 7.00) | 0.3 |
| **HbA1c (%)** | median [IQR] | 8.9 [8.1 to 9.9] | 12.6 [10.4 to 13.6] | <0.001* |
| **BMI Percentile** | median [IQR] | 74 (42, 92) | 59 (29, 78) | 0.036* |
| **Metabolic Control** | Controlled | 14 (11%) | 0 (0%) | 0.002* |
|  | Uncontrolled | 110 (89%) | 45 (100%) |  |

***Table 5:*** *Uni- and Multivariable logistic regression model for periodontitis.*

| Risk factor | Unadjusted OR (95% CI) | p-value | Adjusted OR (95% CI) | p-value | |
| --- | --- | --- | --- | --- | --- |
| Age (years) | 1.21 (0.9 to 1.35) | <0.001* | 1.23 (1.1 to 1.41) | 0.002* | |
| Mean BMI (percentile) | 0.3 (0.1 to 0.93) | 0.037* | 0.32 (0.09 to 1.05) | 0.06 | |
| Duration of DM (years) | 1.04 (0.93 to 1.15) | 0.5 | 0.95 (0.83 to 1.07) | 0.4 | |
| Puberty-Yes | 1.76 (0.88 to 3.58) | 0.11 | 1.06 (0.48 to 2.39) | 0.9 | |
| Sex-Male | 1.33 (0.67 to 2.67) | 0.4 | 1.29 (0.62 to 2.71) | | 0.5 |
| HbA1c (%) | 2.28 (1.81 to 3.01) | <0.001* | 2.38 (1.83 to 3.23) | | <0.001* |

*Adjusted for Age, HbA1c, BMI, and duration of DM and puberty*

** Statistically significant*
